# Refining Planning for Stereoelectroencephalography: A Prospective Validation of Spatial Priors for Computer-Assisted Planning With Application of Dynamic Learning

**DOI:** 10.3389/fneur.2020.00706

**Published:** 2020-07-17

**Authors:** Vejay N. Vakharia, Rachel E. Sparks, Alejandro Granados, Anna Miserocchi, Andrew W. McEvoy, Sebastien Ourselin, John S. Duncan

**Affiliations:** ^1^Department of Clinical and Experimental Epilepsy, University College London, London, United Kingdom; ^2^National Hospital for Neurology and Neurosurgery, London, United Kingdom; ^3^Chalfont Centre for Epilepsy, London, United Kingdom; ^4^School of Biomedical Engineering and Imaging Sciences, King's College London, London, United Kingdom

**Keywords:** stereoelectroencephalography, EpiNav, computer-assisted planning, machine learning, spatial priors, epilepsy surgery

## Abstract

**Objective:** Stereoelectroencephalography (SEEG) is a procedure in which many electrodes are stereotactically implanted within different regions of the brain to estimate the epileptogenic zone in patients with drug-refractory focal epilepsy. Computer-assisted planning (CAP) improves risk scores, gray matter sampling, orthogonal drilling angles to the skull and intracerebral length in a fraction of the time required for manual planning. Due to differences in planning practices, such algorithms may not be generalizable between institutions. We provide a prospective validation of clinically feasible trajectories using “spatial priors” derived from previous implantations and implement a machine learning classifier to adapt to evolving planning practices.

**Methods:** Thirty-two patients underwent consecutive SEEG implantations utilizing computer-assisted planning over 2 years. Implanted electrodes from the first 12 patients (108 electrodes) were used as a training set from which entry and target point spatial priors were generated. CAP was then prospectively performed using the spatial priors in a further test set of 20 patients (210 electrodes). A K-nearest neighbor (K-NN) machine learning classifier was implemented as an adaptive learning method to modify the spatial priors dynamically.

**Results:** All of the 318 prospective computer-assisted planned electrodes were implanted without complication. Spatial priors developed from the training set generated clinically feasible trajectories in 79% of the test set. The remaining 21% required entry or target points outside of the spatial priors. The K-NN classifier was able to dynamically model real-time changes in the spatial priors in order to adapt to the evolving planning requirements.

**Conclusions:** We provide spatial priors for common SEEG trajectories that prospectively integrate clinically feasible trajectory planning practices from previous SEEG implantations. This allows institutional SEEG experience to be incorporated and used to guide future implantations. The deployment of a K-NN classifier may improve the generalisability of the algorithm by dynamically modifying the spatial priors in real-time as further implantations are performed.

## Introduction

Stereotactic neurosurgery requires precise pre-operative trajectory planning and accurate implementation to ensure safety and efficacy. Stereoelectroencephalography (SEEG) is a diagnostic procedure in which multiple electrodes, typically 10–16, are implanted within the brain in patients with drug-refractory focal epilepsy to approximate the epileptogenic zone so that subsequent resective or ablative interventions can render the patient seizure-free. The most significant risk of this procedure is intracerebral hemorrhage, which results in significant morbidity in 2–3% of cases (1). Various surgical techniques are employed for insertion of SEEG electrodes including frame-based, frameless and robotic methods with mean target point accuracies of 2–3 mm (2, 3). To maximize safety, surgeons plan SEEG trajectories to maximize distance from vasculature. Other important considerations include accurate targeting of the regions of interest (ROIs), avoidance of critical structures, maximizing gray-matter sampling, orthogonal drilling angles to the skull, avoidance of other electrodes, optimal spatial sampling of the putative epileptogenic zone and minimizing intracerebral trajectory length. Various computer-assisted planning (CAP) algorithms have been employed to optimize these factors. EpiNav™ is one such stereotactic planning platform that has been applied to SEEG (4–6), laser interstitial thermal therapy (7, 8) and tumor biopsy (9). Previous studies have shown external blinded feasibility ratings of CAP generated trajectories were not significantly different from expert manually planned trajectories, yet due to the wide variation in individual surgeon's planning preferences, these were 62 and 69%, respectively (5). Another potential reason for this is the reliance on whole-brain parcellations to constrain the entry and target points, which in many cases are large structures that require multiple electrodes to pass through them. Furthermore, the algorithms are static without the ability to adapt or learn from previous trajectory planning experience.

Here we present the most extensive series to date of patients that have undergone prospective SEEG planning with CAP. We provide spatial priors to augment CAP by learning from the first 12 patients as a “training set” and subsequently applying this to the prospective planning of a further 20 patients as a “test set.” To aid in the generalisability of the algorithm, we additionally utilize K-nearest neighbor (K-NN) clustering as an active learning algorithm to dynamically modify the priors based on individual surgeon's planning preferences.

## Methods

### Patient Inclusion

A total of 32 patients (17 male) with drug-resistant focal epilepsy, in whom SEEG was performed as part of their routine care at The National Hospital for Neurology and Neurosurgery, London, U.K., were included in this prospective validation study. Patients underwent SEEG implantation between February 2017 and March 2019.

All patients underwent a standardized multi-disciplinary assessment consisting of specialist input from neurologists, neurosurgeons, neurophysiologists, neuropsychologists, and psychiatrists. SEEG trajectory target selection was based on an estimation of the seizure onset zone derived from a review of all pre-surgical investigations, including the clinical history and semiology, scalp EEG/video telemetry, neuropsychological and neuropsychiatric evaluations, structural, and functional MRI, PET, and SPECT imaging. Entry regions were also specified for SEEG trajectories where the lateral neocortex was also of electrophysiological interest.

### Ethical Approval

Ethical approval for this study was provided by the National Research Ethics Service Committee London, approval reference: 12/LO/0377. Written consent was obtained from all patients before inclusion in the study.

### EpiNav™

Pre-operative SEEG planning was performed within the EpiNav™ platform (Center for Medical Imaging Computing, University College London/King's College London) which has been described previously (4). In brief, a single gadolinium-enhanced T1 acquisition is used as a reference image to which all other imaging modalities are registered. A whole-brain parcellation was generated, using Geodesic Information Flow version 3.0 (GIF) (10), from which models of the cortex, gray matter and sulci are extracted in an automated fashion. Vascular segmentations were performed following application of a Sato filter to the pre-operative digital subtraction angiography and manual thresholding (11). Digital subtraction angiography was performed 1–2 weeks before SEEG implantation under local anesthesia in the biplanar angiography suite. Depending on the spatial distribution of the SEEG implantation and the patient's individual anatomy, injections of the ipsilateral internal carotid artery and a vertebral artery were performed. The EpiNav™ algorithm generates SEEG trajectories based on optimization of user-defined parameters, which include intracerebral length, drilling angle to the skull, gray matter sampling ratio, minimum distance from vasculature, risk score, and avoidance of critical structures (12). For an in-depth discussion on planning parameter selection see (13). The user-defined parameters applied during this study are shown in Table 1.

**Table 1:** Computer-assisted planning parameters.

**Parameter**	**Value**
Intracerebral length (mm)	<90
Drilling angle to the skull (deg)	<30 to orthogonal
Gray matter sampling ratio	Maximize
Minimum distance from vasculature (mm)	>3
Risk score	<1
Avoidance of critical structures	Superficial sulcal model
	Vascular model
	Basal ganglia/brainstem
	Frontal and occipital horns of the lateral ventricles.
Distance between electrodes (mm)	>10

The risk score is a mathematical representation of the size of the avascular corridor through which the planned trajectory passes in order to reach the target. It is calculated by fitting 128 nodes along the planned trajectory and measuring the distance between the trajectory and vasculature at each node (4, 14). A cumulative score is then provided scaled by the minimum distance defined by the user. In this study, a 3 mm minimum distance from vasculature was applied, resulting in trajectories that pass within 3 mm of a vessel returning a risk score >1. The 3 mm safety margin is a user-defined setting within the software that can be altered based on the planning preferences of the neurosurgeon. Based on our previous implantation accuracies (15) and the recommendations of Cardinale et al. (16), we calculate the minimum permissible distance from vasculature using the following equation:

$$\begin{array}{l} Safety\;Margin\;\left(mm\right)=Electrode\;radius\;\left(mm\right)+\frac{\Sigma \|i-î\|}{n}\\ \;+3\sigma \end{array}$$

where, $\frac{\Sigma \|i-î\|}{n}$ represents the mean implantation error and σ the standard deviation of the implantation error.

**Figure 1:**
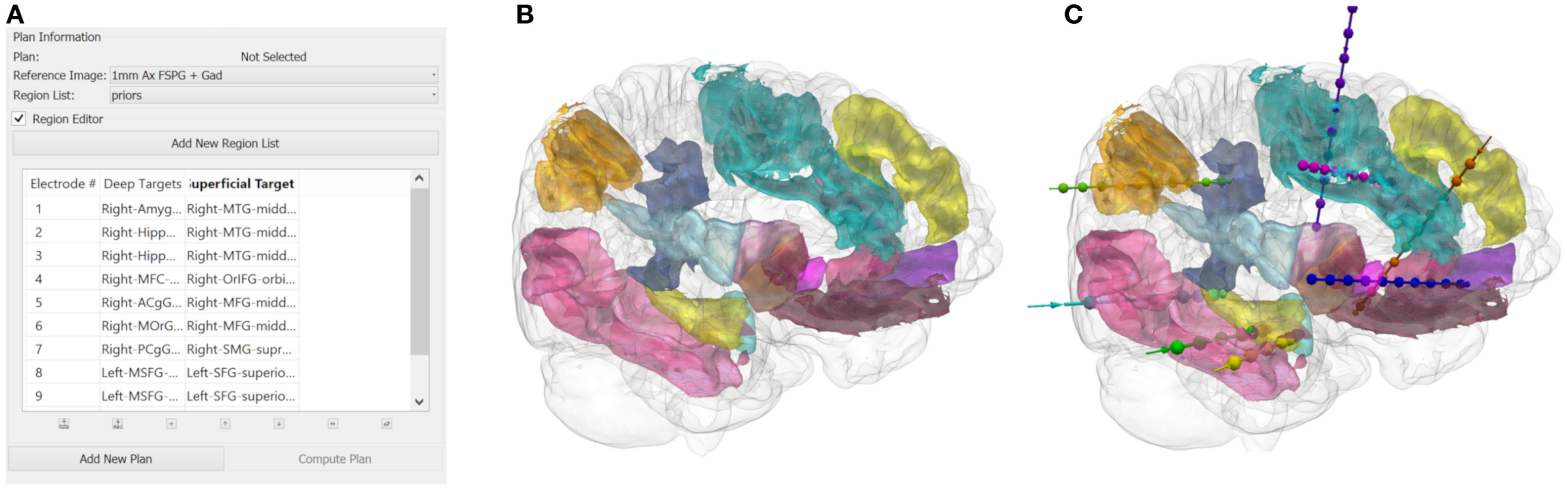
**(A)** A illustrative example of an anatomy-driven multiple trajectory planning strategy (3), with the target and entry points for the trajectory specified by the user. **(B)** The 3D segmentation of the whole brain structures outlined in the strategy and **(C)** the corresponding CAP trajectories optimizing for the user-defined parameters.

The user inputs the implantation strategy by typing or selecting the anatomical ROIs. The entry and target regions are based on the segmentation provided by the GIF parcellation. For pragmatic purposes, we define the entry region as the most superficial anatomical structure through which the trajectory enters the brain and the target region as the deepest point of the trajectory. The vector between the entry and target point defines the trajectory vector. We stress that during SEEG procedures all gray matter contact points along the trajectory are considered target structures and hence we extend implantations to deep structures so that as much information as possible can be gained from each implanted electrode. Constraining the automated planning algorithm to the target region alone allows the global minima to be identified for that target region whereas the additional constraint of the entry region returns the local minima. An example of a typical strategy and plan generated from the GIF parcellation is shown in Figure 1. The automated planning algorithm first removes trajectories that do not adhere to the length and angle constraints. Next, trajectories that do not pass through the entry region, if specified, or conflict with critical structures are also removed. The remaining trajectories are then optimized for gray matter sampling and returned to the user in a risk-stratified manner, i.e., lowest risk first. For a more detailed description of the computer-assisted planning algorithm please see (4, 6).

After CAP, the user reviews each trajectory to ensure clinical feasibility and safety. The potential trajectories generated for a specific target (or entry-target pair) can be iterated through using the “Next Entry” or “Next Target” functions. Manual changes to the entry and target points can also be performed by the user if no suitable CAP generated trajectory is found.

### Cluster Generation

Following prospective SEEG planning and surgical implantation in the first 12 cases (108 electrodes), each patient's reference image was normalized to the MNI-152 (ICBM 2009a Non-linear Asymmetric) group template (17). The parameters for transformation were then applied to the electrode trajectories and coordinate points for the entry and target points were extracted. Right and left side trajectories were combined through flipping. Entry point coordinates were taken at the intersection of the planned trajectory and the cortical surface. The cluster centroids for trajectories were calculated from the coordinates in cases in which the ROI was targeted five or more times to form the training set. Trajectories targeting patient-specific abnormalities, such as lesions or PET/SPECT abnormalities were excluded as these were not generalizable. A total of 13 entry and 14 target ROIs were included. Within-cluster sum of squares (WCSS) was calculated to quantify the extent of variance. Based on the normalized trajectories, spatial priors were then generated to constrain the entry and target points.

### Prospective Validation

Prospective planning was performed in a further 20 patients (210 electrodes) with spatial priors derived from the training set. The predictive utility of the spatial priors was determined by the proportion of trajectories that passed through both the entry and target priors. In addition, the Euclidean distances between the cluster centroids from the prospective trajectories (test set) and those derived from the first 12 cases (training set) were calculated.

### Adaptive Learning

We also implemented a system whereby spatial priors could adapt to evolving SEEG planning practices. The added flexibility would allow the priors to adapt and potentially incorporate new entry or target points outside of the original priors. This would permit external institutions to use the above spatial priors as a starting point and, with subsequent SEEG implantations, enable it to adapt to the individual surgeon's or institutional preferences. This was accomplished through the implementation of a K-Nearest Neighbor (K-NN) classifier to the prospective validation dataset. The K-NN was deployed using Euclidean distance from 5 uniformly weighted neighbors to determine the classifier assignments.

Computational analysis was performed with custom scripts utilizing functions from the following python libraries: Pandas, Numpy and SciKit learn. The Matplotlib library was used for data visualization.

## Results

### Priors Validation

**Figure 2:**
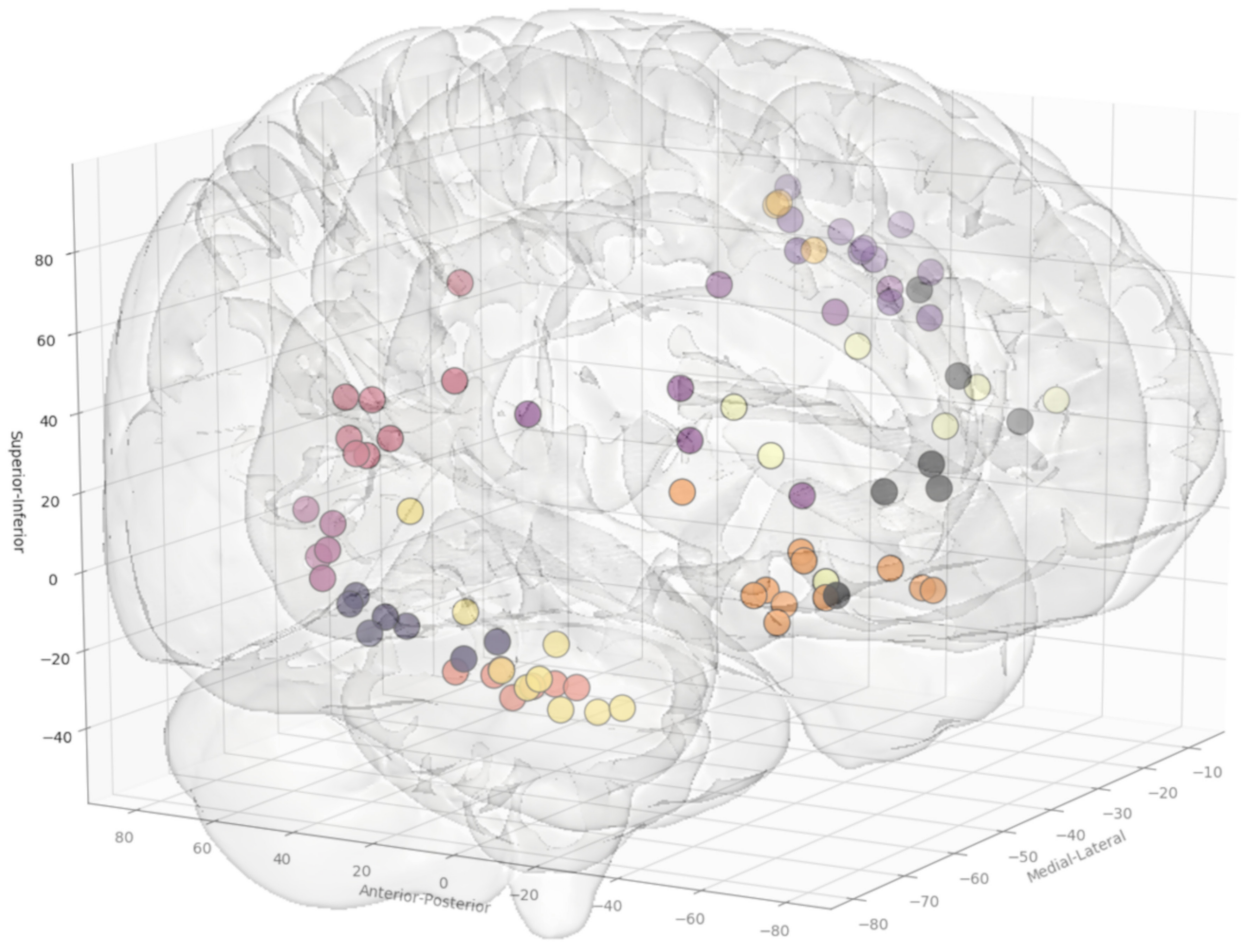
Coordinates of the entry points, shown from a right anterolateral projection for electrode trajectories within the training set (*n* = 12 patients). Table 2 outlines the ROIs included for the entry and target points. Greater transparency represents trajectory points closer to the midline.

**Figure 3:**
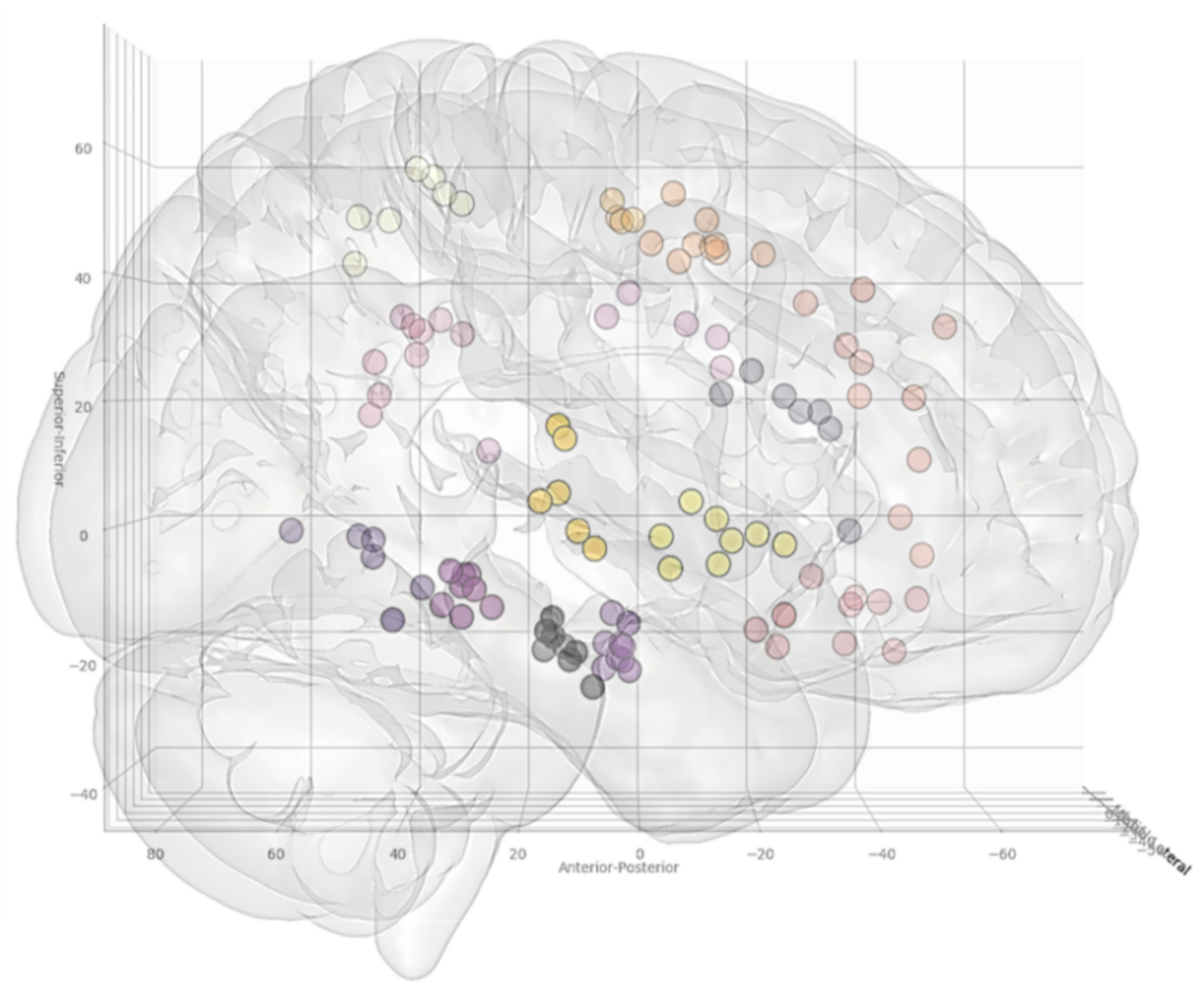
Coordinates of the target points, shown from a right lateral projection, for electrode trajectories within the training set (*n* = 12 patients). Table 2 outlines the ROIs included for the entry and target points. Greater transparency represents trajectory points closer to the midline.

In total, 13 entry and 14 target point clusters were included in the training set derived from the first 12 patients (Figure 2). An entry prior for the posterior insula was not generated due to the wide dispersion of selected entry points beyond that of a single GIF parcellation ROI, indicating a lack of consistency during planning. An overview of color coded priors derived from the entry and target regions of the training set are shown in Figure 3.

**Figure 4:**
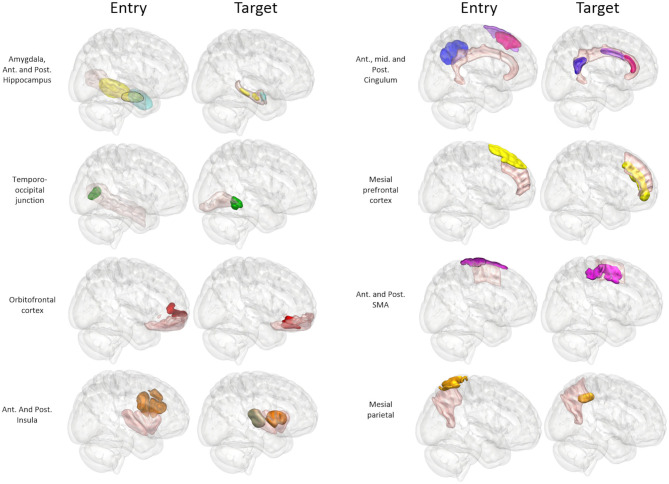
Panel of 3D images shown from right lateral projection with color-coded entry (columns 1 and 3) and target (columns 2 and 4) priors within GIF defined anatomical regions (pink). Color scheme: Amygdala: Cyan, Hippocampus: Yellow, Temporo-occipital junction: Green, Orbitofrontal cortex: Red, Anterior Insula: Brown, Posterior Insula: Gray, Anterior Cingulum: Dark pink, Middle Cingulum: Purple, Posterior Cingulum: Blue, Mesial prefrontal cortex: Yellow, Supplementary sensory-motor area: Magenta, Precuneus: Orange.

**Figure 5:**
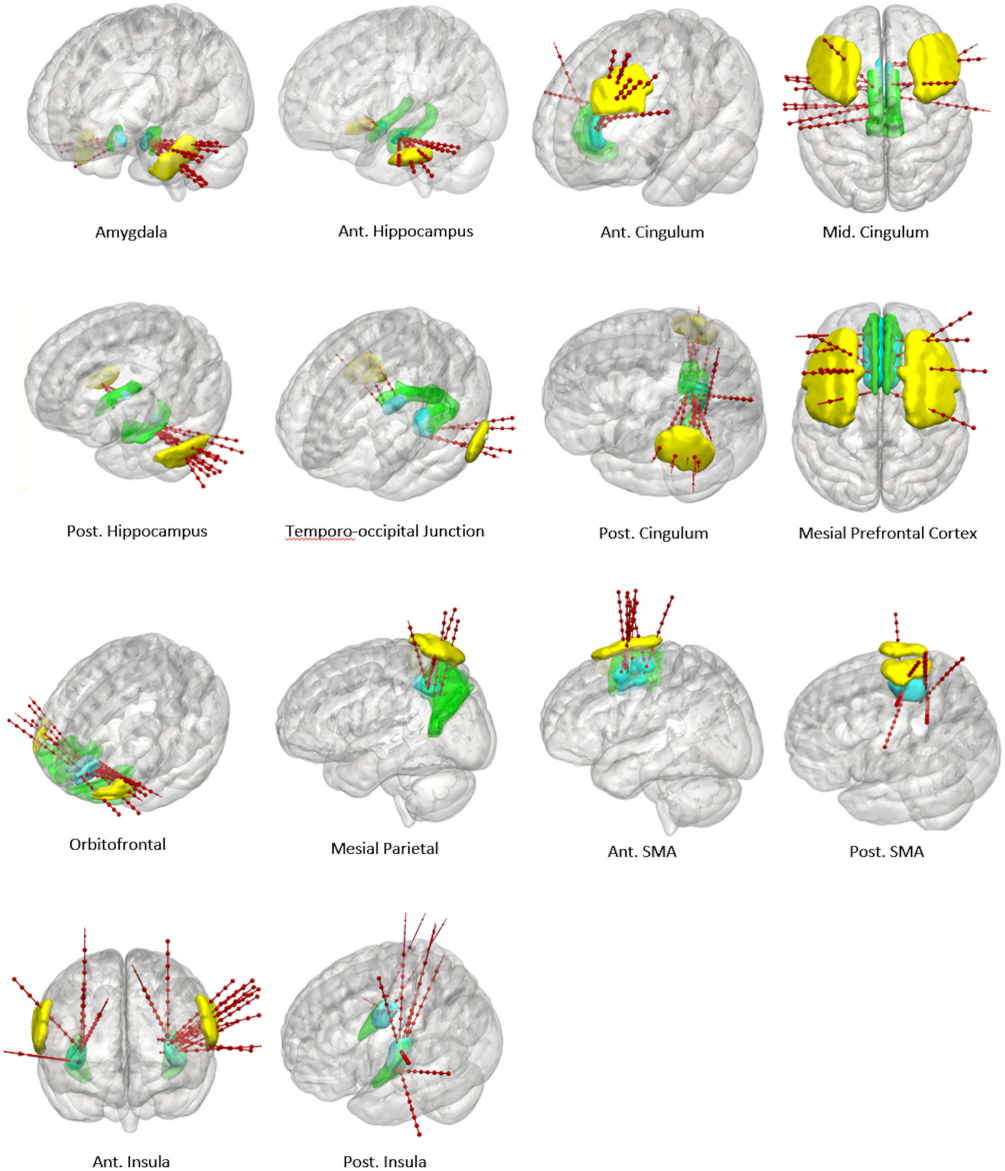
Panel of 3D cortical images shown from various projections with implanted electrode trajectories from the test set (red) passing through the entry priors (yellow) and the target priors (blue) derived from the training set. GIF defined anatomical structures are shown in green.

A further 20 patients were then prospectively planned and implanted using the spatial priors derived from the previously implanted trajectories within the training set. Of the prospectively planned trajectories, 79% (129/163) were able to be planned and implanted using the spatial priors to restrict the entry and target regions (see Table 2 and Figure 4). The remaining 21% (34/163) of prospectively implanted trajectories required entry or target points outside of these priors (see Figure 5). All prospectively planned and implanted trajectories sampled the intended ROIs and there were no postoperative complications or hemorrhages. Coordinates for the entry and target point cluster centroids from the training set and Euclidean distance to the cluster centroid from the prospective group are shown in Supplementary Table 1. On average, the training and test cluster centroids for the majority of entry and target points were 10 mm apart, with the most notable exception being mesial prefrontal cortex electrodes. This most likely reflects the variability of cerebral vasculature between patients and the large anatomical area for electrophysiological sampling.

**Table 2:** Results of implanted computer-assisted planning electrode in relation to the priors.

	**No. trajectories**	**Through prior**	**Outside prior**
Orbitofrontal	15	13 (87%)	2 (13%)
Amygdala	17	16 (94%)	1 (6%)
Anterior hippocampus	11	8 (73%)	3 (27%)
Posterior hippocampus	13	10 (77%)	3 (23%)
Temporo-occipital junction	6	6 (100%)	0 (0%)
Anterior cingulum	10	10 (100%)	0 (0%)
Middle cingulum	13	7 (54%)	6 (46%)
Posterior cingulum	15	12 (80%)	3(20%)
Mesial pre-frontal cortex	9	8 (89%)	1 (11%)
Anterior SSMA	12	11 (92%)	1 (8%)
Posterior SSMA	8	4 (50%)	4 (50%)
Precuneus	7	4 (57%)	3 (43%)
Anterior insula	17	10 (59%)	7 (41%)
Posterior insula	10	10 (100%)	0 (0%)
Total	163	129 (79%)	34 (21%)

### Adaptive Learning

Given that 21% of the prospectively planned trajectories were outside of the spatial priors, a K-NN machine learning classifier was applied to dynamically refine the boundaries of the entry and target priors based on the data in the training set. Subsequent implantations from the test set were then added to the training set data in 5-folds (random selection of 42 new trajectories with each fold). The K-NN classifier was iteratively re-applied and the dynamic changes in the target priors are shown in Supplementary Figure 1.

## Discussion

We have previously shown that CAP using the EpiNav™ platform can optimize trajectory planning parameters and return feasible plans in < one-third of the time required for manual planning (6). Computer-assisted planning, however, requires familiarity with the software and algorithms as well as multimodal image processing. In the present study, we undertake a prospective validation of spatial priors to further refine CAP for SEEG electrode trajectories by removing the reliance on ROIs derived from whole-brain parcellations. Based on a training set of 12 patients (108 electrodes) in which the EpiNav™ platform was used for CAP utilizing the GIF parcellation, we generated entry and target spatial priors for common ROIs that were targeted five or more times. Five was chosen as an arbitrary threshold to allow accurate morphological delineation and cluster centroid calculation for the prior. The spatial priors were then prospectively used to restrict the entry and target points instead of the GIF parcellation for CAP in a further 20 patients (210 electrodes). The incorporation of spatial priors allowed feasible trajectories to be returned for 79% of the electrodes. Each of these was subsequently implanted into patients without complication. For the remaining 21%, the implemented entry or target points were outside of the spatial priors. A machine learning classifier was implemented to dynamically modify the priors to account for this. We provide the spatial priors in MNI template space for use by other institutions during CAP or manual planning and as a potential starting point for standardization of SEEG trajectories.

This is the first prospective study describing the utility of spatial priors to refine computer-assisted SEEG trajectory planning. Two main methods for SEEG CAP have been implemented in the literature. The first is where the user defines a target point and the algorithm returns a trajectory with the lowest risk score (18). This has the benefit of ensuring that the precise ROI within the anatomical structure is targeted, but this limits the algorithm to return the local, but not global, minimum risk score. It may also lead to a failure of the CAP algorithm to return a feasible trajectory, especially if the chosen target point is adjacent to a critical structure and therefore contravenes a “hard constraint” within the planning algorithm. Due to this, some groups suggest “roughly” selecting the entry and target point (19–21). The algorithm then returns trajectories within a 1 cm radius allowing for slightly more variation in the entry and target points. This method still requires manual user interaction for rough placement. Another method that has been implemented is to allow the algorithm to define the entry and target points automatically within predefined anatomical structures (4). This is reliant on the anatomical segmentation provided by whole-brain parcellations such as Freesurfer (22) or GIF (10). In general, whole-brain parcellations are developed from healthy controls and the accuracy of the segmentation may fail in patients with gross anatomical abnormalities or following previous surgery. Another limitation is that in some cases the anatomically defined entry and target regions may be very large such as electrodes targeting the anterior cingulum, which typically enter through the middle frontal gyrus. The computer planning algorithm then returns the global minimum risk score, but this may not be practical or feasible. Algorithms have been able to counter this problem to some extent through maximizing spatial distribution but only when multiple electrodes pass through a single ROI (4). One example of this can be seen with temporal implantations. In such a scenario sampling the temporal pole, amygdala, anterior hippocampus, posterior hippocampus and temporo-occipital junction may be required. Unless the clinical scenario dictates otherwise, it is likely that that entry points for all of these electrode trajectories will pass through the middle temporal gyrus. It is beneficial, therefore for the lateral neocortical sampling to be spatially distributed along the anteroposterior axis to prevent electrode conflicts and also aid in the delineation of the lateral neocortical resection margins. More advanced systems also enable the user to iterate through the proposed trajectories in a risk-stratified manner until a feasible trajectory with the lowest risk score is identified (5). Spatial priors can overcome this limitation as the entry and target points are confined to previously implemented trajectories. This removes the reliance on whole-brain parcellations for the entry and target point constraints and ensures reliable spatial sampling. Another benefit is that the risk-stratified trajectories returned to the user are more likely to be clinically acceptable and reduces the need to iterate through the options. In generating these priors, we purposefully excluded trajectories that targeted unique patient-specific abnormalities, such as focal cortical dysplasia, as these would not be generalizable when considering trajectory planning in other patients. In such cases, computer-assisted planning can still be utilized using the segmentation of the lesion as the target and allow the algorithm to choose the most appropriate entry point (12).

As a further analysis, we implemented a K-NN classifier as part of an adaptive learning algorithm. Here the K-NN classifier was used to generate the boundaries that define the priors for the entry and target points of the electrodes in the training set. Electrode entry and target points were then iteratively added in five equal folds, each with randomly selected trajectories. The K-NN classifier then adjusted the priors based on the additional feasible electrode information. The unique benefit of this adaptive technique is the ability to dynamically adapt to changing planning preferences and learn evolving individual surgical preferences. In this implementation, the weighting was uniformly distributed, in that the entry and target points contributed to the classifier equally. Where surgeons prefer entry or target regions within a specific location, weightings could also be applied to favor the distribution. Machine learning has previously been applied retrospectively to SEEG trajectory analysis to identify stereotyped implantation schema (23). In this work, the authors reviewed previous manually planned trajectories from their institution and used a K-NN clustering algorithm to identify that their implantation practices would be distilled down to 8 unique strategies. This work adds further utility as a potential recommendation system i.e., where the algorithm can identify predefined electrode trajectories and suggests where further electrodes are needed. The authors then show that the manually implanted trajectories can be further optimized by applying their computer-assisted planning pipeline once the surgeon has roughly placed an entry and target point within a 1 cm vicinity. It is unclear if these stereotype implantations are generalizable to other institutions that have varying practices. The automated trajectories in their study have also not been prospectively implanted in patients and hence there is no clinical validation of the true safety of the automated trajectories. The work presented in this manuscript, however, is distinct for the following reasons. Firstly, we make no suggestions regarding which targets are included in the implantation strategy as this is defined following the multidisciplinary review of the presurgical evaluation. Instead, we focus on improving the reliability, efficiency and adaptability of precise electrode planning. Secondly, all of the automated trajectories were prospectively implanted in our series without complication. Thirdly, the spatial priors generated from the training set leverages our institutional experience and the active learning approach mimics the real-world use of the platform if an external center were to add their implantation trajectories. Additionally, the spatial priors no longer require an anatomical segmentation atlas once generated.

There are limitations to this study. The entry and target priors are derived from a single-center incorporating planning practices from two surgeons. To mitigate this, a K-NN adaptive learning technique was implemented to dynamically modify the priors based on varying surgeon and institutional practices. We also found a considerable variation in the entry points relating to the posterior insula trajectories preventing the generation of an entry prior that was more constrained than the GIF parcellation. The principal reason for this was the between-patient variability in oblique vs. orthogonal (transsylvian) trajectories as a result of vascular constraints. In this study, a K-NN classifier was chosen over other potential learning algorithms as it allows for 3-dimensional clustering in a discriminative non-parametric fashion. Further work should also focus on evaluating other machine learning classifiers. Finally, the spatial prior and prospectively implanted trajectories are based on the pre-operative acquisition of a DSA to guide SEEG trajectory planning, as this is the standard of care at the study institution. The priors are equally applicable, however, to centers that do not use DSA for planning as CAP can be performed with any vascular imaging modality, but conflicts with non-segmented vasculature may be more frequent depending on the minimum clinically significant vessel size considered (24).

## Conclusion

Spatial priors are a valuable contribution to CAP, allowing future implantations to be guided by previous planning experience. Through the prospective application of spatial priors, we show that feasible trajectories can be planned and implanted in test cases enabling CAP to be performed without the reliance on whole-brain parcellations. In addition, experience from SEEG trajectory planning can be continually refined and used to update the spatial priors dynamically, through the implementation of a K-NN classifier. This opens the possibility of the algorithm adapting to evolving practices as well as dynamically learning individual surgeon's planning preferences from subsequent implantations. Future work will focus on validating this novel preliminary approach through external, multi-center SEEG implantation data.

## Data Availability Statement

All summary datasets generated in this study are included in the article/Supplementary Material.

## Ethics Statement

The studies involving human participants were reviewed and approved by National Research Ethics Service Committee London, approval reference: 12/LO/0377. The patients/participants provided their written informed consent to participate in this study.

## Author Contributions

VV was involved with the conception, design, data acquisition, and analysis as well as manuscript production. All authors critically reviewed the final manuscript prior to submission, contributed to the article, and approved the submitted version. JD was involved with the study design and provided study oversight. SO was involved with computer programing aspects of the EpiNav platform and provided study oversight. AWM was involved with data acquisition. AM was involved with data acquisition. AG was involved with the data acquisition and computer programing aspects of the EpiNav platform. RS was involved with the data acquisition and computer programing aspects of the EpiNav platform.

## Conflict of Interest

The authors declare that the research was conducted in the absence of any commercial or financial relationships that could be construed as a potential conflict of interest.

## Supplementary Material

Supplementary Figure 1 MNI coordinate system: K-NN classifier used to define spatial prior boundaries for target points based on the training set and subsequent addition of 5-folds of data from the test set. Dynamic refinement of the spatial priors can be seen with addition of subsequent target point information.

Supplementary Table 1 Coordinates of training and test set electrode cluster centroids in MNI space.

## Abbreviations

CAP: Computer-assisted planning
K-NN: K-nearest neighbor
PET: Positron emission tomography
ROI: Regions of interest
SEEG: Stereoelectroencephalography
SPECT: Single-photon emission computer tomography
WCSS: Within-cluster sum of squares.
