# Supplementary figures and images for: Refining Planning for Stereoelectroencephalography: A Prospective Validation of Spatial Priors for Computer-Assisted Planning With Application of Dynamic Learning
Source: Front Neurol. 2020 Jul 17;11:706. doi: 10.3389/fneur.2020.00706 (PMC7380116; doi:10.3389/fneur.2020.00706)

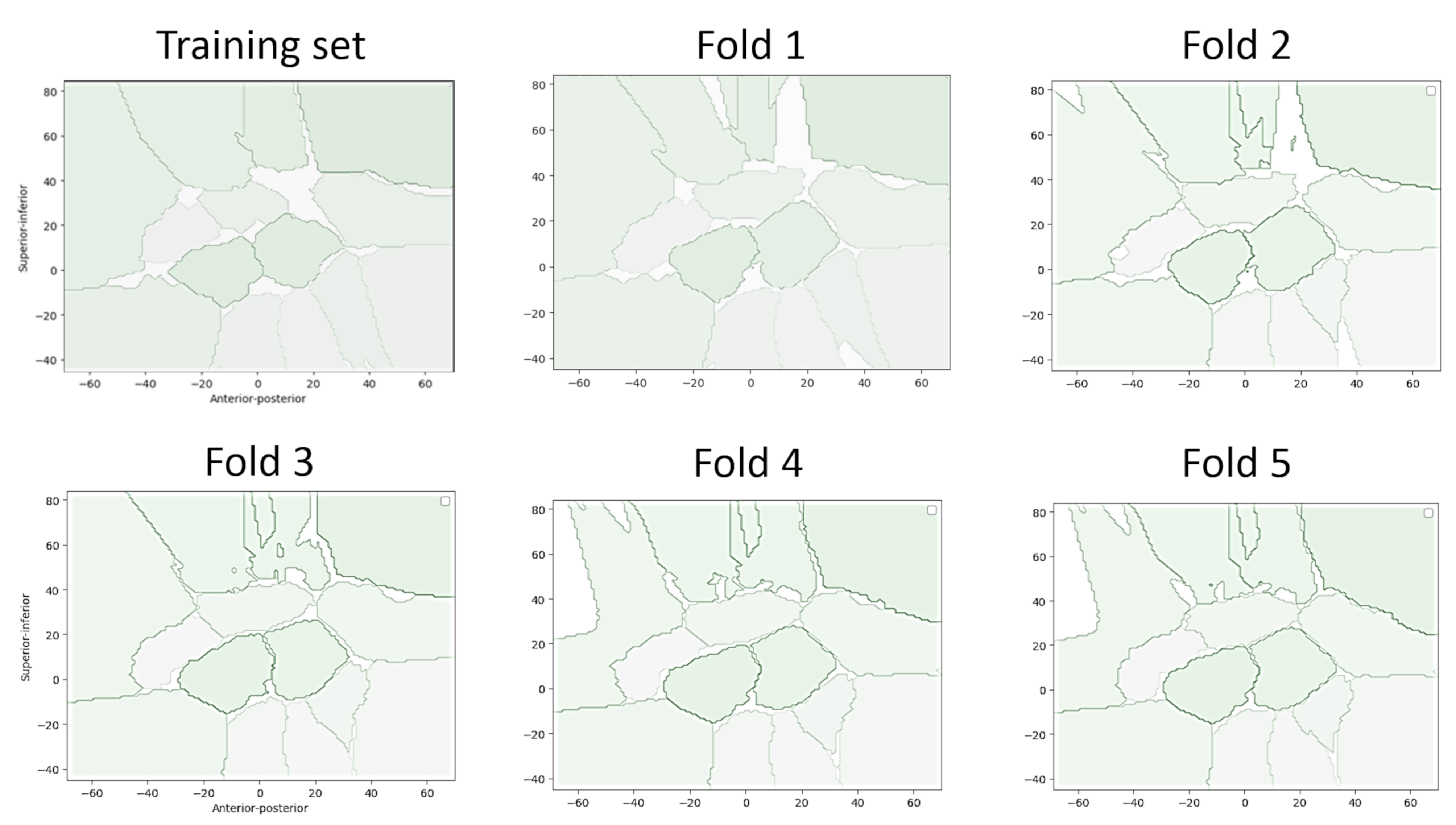

Supplement: Supplementary Figure 1 — MNI coordinate system: K-NN classifier used to define spatial prior boundaries for target points based on the training set and subsequent addition of 5-folds of data from the test set. Dynamic refinement of the spatial priors can be seen with addition of subsequent target point information. [file Image_1.TIF]
