# Supplementary material for: Refining Planning for Stereoelectroencephalography: A Prospective Validation of Spatial Priors for Computer-Assisted Planning With Application of Dynamic Learning
Source: Front Neurol. 2020 Jul 17;11:706. doi: 10.3389/fneur.2020.00706 (PMC7380116; doi:10.3389/fneur.2020.00706)
Supplement: Supplementary Table 1 — Coordinates of training and test set electrode cluster centroids in MNI space. [file Table_1.docx]

Supplementary Table 1:

|  | | Entry | | | | Target | | | |
| --- | --- | --- | --- | --- | --- | --- | --- | --- | --- |
|  | | Cluster Centroid Coordinates* | | | Test set error | Cluster Centroid Coordinates* | | | Test set error |
| ROI | Method | X | Y | Z | Euclidean (mm) | X | Y | Z | Euclidean (mm) |
| Amygdala | Training | ±62.63 | 9.47 | -22.41 | 10.16 | ±14.29 | 3.55 | -21.05 | 2.49 |
|  | Test | ±57.40 | 2.24 | -27.27 | - | ±15.06 | 5.53 | -19.74 | - |
| Anterior Hippocampus | Training | ±66.20 | 8.00 | -26.07 | 13.59 | ±20.44 | 13.33 | -20.66 | 4.25 |
|  | Test | ±69.07 | 18.68 | -18.19 | - | ±21.16 | 14.75 | -16.73 | - |
| Posterior Hippocampus | Training | ±69.12 | 36.34 | -11.19 | 6.53 | ±23.53 | 30.35 | -11.13 | 4.29 |
|  | Test | ±69.87 | 36.35 | -9.50 | - | ±25.17 | 26.46 | -11.89 | - |
| Temporo-occipital junction | Training | ±67.41 | 55.60 | 3.71 | 10.29 | ±13.59 | 47.81 | -6.84 | 9.09 |
|  | Test | ±61.63 | 51.20 | -3.57 | - | ±14.58 | 40.97 | -12.73 | - |
| Orbitofrontal cortex | Training | ±51.96 | -46.41 | 0.08 | 6.52 | ±2.05 | -35.08 | -16.66 | 5.23 |
|  | Test | ±48.19 | -43.82 | 4.72 | - | ±5.77 | -34.26 | -13.08 | - |
| Anterior Cingulum | Training | ±38.87 | -47.14 | 32.07 | 18.08 | ±3.02 | -28.10 | 16.48 | 3.64 |
|  | Test | ±46.12 | -31.09 | 36.19 | - | ±2.49 | -26.58 | 13.22 | - |
| Middle Cingulum | Training | ±46.65 | -20.74 | 46.22 | 8.57 | ±1.62 | 5.49 | 29.48 | 9.75 |
|  | Test | ±45.40 | -14.19 | 51.59 | - | ±2.34 | -3.52 | 33.15 | - |
| Posterior Cingulum | Training | ±65.10 | 41.82 | 39.26 | 9.27 | ±1.95 | 41.16 | 27.60 | 3.37 |
|  | Test | ±60.53 | 33.91 | 37.63 | - | ±2.49 | 38.44 | 25.67 | - |
| Mesial prefrontal cortex | Training | ±35.38 | -54.05 | 26.89 | 28.80 | ±2.91 | -44.27 | 20.73 | 15.90 |
|  | Test | ±24.59 | -38.85 | 48.83 | - | ±2.65 | -31.24 | 29.84 | - |
| Anterior SSMA | Training | ±20.87 | -19.80 | 66.95 | 18.65 | ±3.36 | -9.94 | 47.31 | 10.76 |
|  | Test | ±33.90 | -6.46 | 66.90 | - | ±3.49 | 0.18 | 50.94 | - |
| Posterior SSMA | Training | ±23.90 | -5.70 | 74.89 | 19.48 | ±3.58 | 2.80 | 51.37 | 11.41 |
|  | Test | ±35.67 | 9.07 | 70.12 | - | ±4.02 | 14.20 | 51.12 | - |
| Mesial parietal | Training | ±35.50 | 52.00 | 71.26 | 8.37 | ±3.70 | 41.29 | 52.61 | 15.31 |
|  | Test | ±41.96 | 50.50 | 66.15 | - | ±2.92 | 49.34 | 39.62 | - |
| Anterior Insula | Training | ±58.26 | -16.00 | 24.83 | 12.07 | ±34.75 | -13.33 | -2.17 | 7.72 |
|  | Test | ±48.50 | -22.97 | 26.17 | - | ±31.64 | -12.98 | 4.88 | - |
| Posterior Insula | Training | - | - | - | - | ±36.87 | 12.47 | 5.72 | 4.93 |
|  | Test | - | - | - | - | ±32.23 | 13.82 | 4.76 | - |

*Coordinates provided in MNI-152 template space

Please note, in MNI space coordinates relating to the X-axis positive values denote the right hemisphere and negative values the left.
